# Improved survival of patients with coronary artery disease and low ejection fraction with ICD implantation versus conventional therapy in a real world survey

**DOI:** 10.1186/1756-0500-5-382

**Published:** 2012-07-27

**Authors:** Timo Aschenbrenner, Johannes Brockmeier, Peter Bramlage, Rolf Fimmers, Alessandro Cuneo, Stefan Hochreuther, Claudia Zemmrich, Ulrich Tebbe

**Affiliations:** 1 Department I - Cardiology, Angiology, Intensive Care Medicine, Klinikum Lippe-Detmold, Röntgenstrasse 18, 32756, Detmold, Germany; 2 Institut für Pharmakologie und präventive Medizin, Mahlow, Germany; 3 Department of Biometry and Medical Statistics, University of Bonn, Bonn, Germany; 4 Abteilung für Kardiologie, Asklepios Klinik St-Georg, Hamburg, Germany

**Keywords:** Implantable cardioverter defibrillator, Cohort study, Myocardial infarction

## Abstract

**Background:**

Coronary artery disease (CAD) is associated with an increased risk for sudden cardiac death. Randomized controlled trials have shown that implantable cardioverter defibrillators (ICD) improve life expectancy unless they are implanted within the first days after an acute myocardial infarction and guidelines recommend their use. We aimed to validate that these results also apply to patients of a typical community hospital in Germany.

**Methods:**

This was a retrospective analysis of patients undergoing coronary angiography in the Lippe-Detmold Hospital between 2003 and 2006. They had to have significant CAD and an ejection fraction (EF) ≤ 35% and no acute myocardial infarction within 28 days of implantation and no history of ventricular fibrillation.

**Results:**

213 patients were included; 70 of which received an ICD. Patients with an ICD implantation were younger (64.8 ± 9.9 vs. 67.9 ± 9.8 years; p = 0.034), had single vessel CAD more frequently (22.9 vs. 11.2%; p = 0.025) and a lower EF (26.7 ± 6.3 vs. 29.1 ± 4.6%; p = 0.006). Hospital readmissions were comparable between the ICD and the control group (68.6 vs. 72.0%; p = 0.602). ICD therapy was associated with a considerable survival benefit compared to conventional therapy (HR 0.52; 95%CI 0.29-0.93; p = 0.027) in a Cox-Proportional Hazards Regression analysis.

**Conclusions:**

Appreciating the potential limitations of retrospective studies, we found that ICD use was associated with improved survival in patients with significant CAD and an EF <= 35% typical for a large tertiary hospital.

## Background

Coronary artery disease (CAD) is associated with an increased risk for angina, acute myocardial infarction (MI), reduced left ventricular ejection fraction (LVEF), congestive heart failure, and sudden cardiac death [1]. Although considerable progress has been made with respect to improving prognosis [2] approaches utilizing antiarrhythmic pharmacotherapy have been misleading [3-6], and sudden cardiac death remains a major threat to patients wellbeing. MADIT II documented that ICD use was associated with improved survival in CAD patients with prior MI and reduced LV function (EF ≤ 30%) [7] while DINAMIT reported no benefit with respect to overall mortality in patients with ICD implantation within days after an acute myocardial infarction [8].

ICD therapy is recommended by the recent guidelines for the primary prevention of ventricular arrhythmias and sudden cardiac death in patients with LV dysfunction due to prior MI who are at least 40d post-MI and have an LVEF ≤ 30-40%, NYHA II and a good reasonable expectation of survival with a good functional status for more than 1 year (Level of Evidence A) [9]. In clinical practice, ICDs appear to reduce all cause and arrhythmic death to a similar degree as in primary prevention trials [10]. We aimed to validate the results of the aforementioned trial in clinical practice by a retrospective documentation of patients with ICD implantation between 2003 and 2006.

## Methods

This was a retrospective analysis of patients undergoing coronary angiography in the cardiology unit of the Lippe-Detmold Hospital, a tertiary care hospital, between January 8^th^ 2003 and December 18^th^ 2006. Ethic committee approval as well as patient informed consent was not obtained due to the retrospective design of the study.

### Patients

Patients included had undergone coronary angiography because of significant CAD and an EF ≤ 35% on monoplane evaluation and had an indication for the primary prevention of ventricular arrhythmias and sudden cardiac death [9]. Exclusion criteria were acute myocardial infarction less than 28 days ago, known cancer (because of the limited life expectancy), and cardio-pulmonary resuscitation due to ventricular fibrillation.

### Documentation

Follow-up data for a duration of at least 30 months were collected from hospital charts when patients were readmitted or via contact by telephone. The following variables were obtained: date of birth, gender, the presence of CAD and the number of vessels diseased, LVEF, the presence of diabetes and concomitant medication. During follow-up data on readmissions to the hospital and the reasons for referral, the adequacy of shocks and overall duration of survival were gathered.

### Statistics

This data were analyzed with Aabel 20/20 data vision 3® from Gigawiz Ltd Co. Descriptive statistics were applied, based on available data and significance calculated using the two-tailed unpaired t-Test. Kaplan-Meier estimates used to compare survival and the p-value calculated by the log-rank test. Further a proportional hazards model was built considering variables displaying significant differences at baseline (p < 0.05).

## Results

### Patient characteristics

A total of 213 patients (70 receiving an ICD, 143 receiving none, 15.0% female) were included into this analysis (Table 1). Patients in the ICD group were significantly younger, had single vessel CAD more frequently and a lower ejection fraction. Except for the use of statins, which was more frequent in patients receiving an ICD, pharmacotherapy was not different between groups.

**Table 1:** Patients characteristics

	**Control**	**ICD**	**p**
	**(n = 143)**	**(n = 70)**	
**Age in years, mean (±SD)**	67.9 ± 9.8	64.8 ± 9.9	0.034
**Gender, women, n (%)**	26 (18.2 %)	6 (8.6 %)	0.065
**Coronary artery disease (CAD)**			
1-vessel CAD, n (%)	16 (11.2 %)	16 (22.9 %)	0.025
2-vessel CAD, n (%)	41 (28.7)	23 (32.9)	0.531
3-vessel CAD, n (%)	86 (60.1)	31 (44.3)	0.029
**Left ventricular ejection fraction (LVEF), %**	29.1 ± 4.6	26.7 ± 6.3	0.006
LVEF, women, %	29.4 ± 4.5	28.7 ± 7.7	
LVEF, men, %	29.0 ± 4.7	26.5 ± 6.1	
LVEF, 1-vessel CAD, %	29.4 ± 4.2	26.3 ± 7.0	
LVEF, 2-vessel CAD, %	28.7 ± 4.7	25.2 ± 7.1	
LVEF, 3-vessel CAD, %	29.3 ± 4.7	28.1 ± 5.0	
**Diabetes, n (%)**	56 (39.2)	24 (34.3)	0.490
**Pharmacotherapy, n (%)**			
Betablockers	132 (93.0)	68 (97.1)	0.215
ACE inhibitors	110 (77.5)	59 (84.3)	0.245
ARBs	19 (13.4)	6 (8.6)	0.307
Statins	93 (65.5)	57 (81.4)	0.017
Diuretics	125 (88.0)	58 (82.9)	0.303
Antiarrhythmics (Amiodarone)	6 (4.2)	3 (4.3)	0.984
Anticoagulation	51 (35.9)	27 (38.6)	0.706

Legend: ARB, Angiotensin II Receptor Blockers; CAD, coronary artery disease; ICD, implantable cardioverter defibrillator, LVEF, left ventricular ejection fraction; SD, standard deviation.

### Therapy

On follow-up hospital readmissions in the ICD group were comparable to the CT group (Table 2). Reasons were rhythm disorders, ICD related reasons and a decompensation of heart failure, which was more frequent in the ICD group. In the control group there were seven late ICD implants and two patients received a cardiac resynchronization device (CRT-D). In the ICD group one device was upgraded to a CRT-D.

**Table 2:** Complications after inclusion and ICD therapy

**Complications after inclusion**	**Control**	**ICD**	**p**
	**(n = 143)**	**(n = 70)**	
Hospital Admissions, n (%)	103 (72.0)	48 (68.6)	0.602
Rhythm disorders, n (%)	18 (12.6)	12 (17.1)	0.369
Heart failure, n (%)	13 (5.1)	18 (37.1)	<0.001
Late implants			
Time from EF to ICD / CRT (months)	33.1 ± 20.7	2.3 ± 4.4	
Late ICD implantation, n (%)	7 (4.9)	-	
CRT-D implantation, n (%)	2 (1.4)	-	
CRT-D upgrade, n (%)	-	1 (1.4)	
ICD therapy (n = 70)	**Survivor**	**Death**	**p**
	**(n = 56)**	**(n = 14)**	
ICD shock, n (%)	15 (26.8)	4 (28.6)	1.000
Inappropriate* shocks, n (%)	3 (5.4)	3 (21.4)	0.090
No ICD shocks, n (%)	38 (67.9)	9 (64.3)	1.000

Legend: ICD, implantable cardioverter defibrillator; Time from EF to ICD, time between determination of EF (Ejection Fraction) and ICD implantation; CRT-D, cardiac resynchronization device; *as inappropiate those shocks were classified that were not due to ventricular fibrillation.

### Survival

**Figure 1 Probability of survival: 0.0; 0.2; 0.4; 0.6; 0.8; 1.0:**
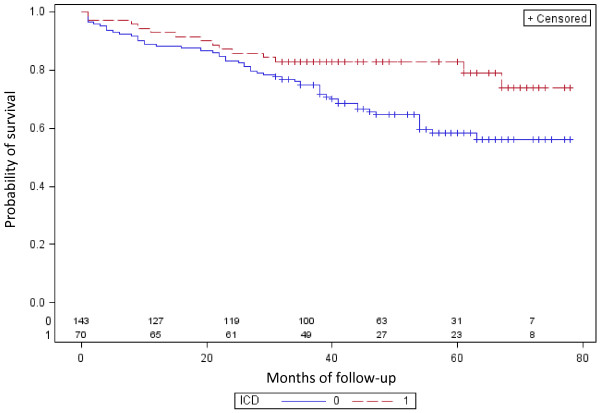
- **Kaplan-Mayer overall survival in patients receiving ICD vs. conventional therapy.** Legend: ICD, implantable cardioverter defirbillator

ICD therapy was associated with a significant survival benefit compared to conventional therapy (HR 0.52; 95%CI 0.29-0.93; p = 0.027) in a Cox-Proportional Hazards Regression analysis. Curves began to diverge at 24 months of follow-up (Figure 1, Table 3). This benefit was preserved in patients with the longest follow-up of 78 months. There were a nominally larger proportion of patients with inappropriate (those not due to ventricular fibrillation) ICD shocks within the group of patients dying (21.4 vs. 5.4%; p = 0.0896, exact fisher test).

**Table 3:** Outcomes of patients in our registry

	**Detmold ICD +**	**Detmold ICD –**
	**(n = 70)**	**(n = 143)**
	Coronary artery disease with an EF ≤ 35 %, no MI within the last 28 days, without known cancer or history of ventricular fibrillation	
		
Mean follow-up	44 months	
Total mortality	20.0	37.8
HR Total mortality	HR 0.52 (95%CI 0.29-0.93); p = 0.027	
Heart failure related hospitalization	25.7	8.3

Legend: HR, hazard ratio; CI, confidence interval, EF, ejection fraction; LV, left ventricular.

## Discussion

Guideline recommendations for the primary prevention of ventricular arrhythmias and sudden cardiac death such as that of the ACC/AHA/ESC [9] rely on information derived from randomized, controlled trials. These trials have a high internal validity for the patient group actually included, but may lack such validity for a substantial proportion of patients in clinical practice. Therefore we aimed to validate these trials in the patient population of our community hospital in Detmold. Overall 213 patients were retrospectively documented and prospectively evaluated for survival and appropriateness of shocks. We found that ICD use was associated with a considerable improvement of survival which was statistically significant.

### Study population

Patients in the present registry were included if they had CAD with an EF ≤ 35% and no MI within the last 28 days. This patient population is only partially present in most of the ICD trials [8,11-15], but has a substantial overlap with those of MADIT-II [7], which included patients with prior MI more than 28 days ago and an EF ≤ 30%. Therefore Table 4 displays clinical characteristics of patients in both MADIT-II and our observation and illustrates that while age, gender and diabetes prevalence was roughly comparable, LVEF was less reduced in our observation (because of the inclusion of patients with an EF ≤ 35 vs. ≤ 30%). Diuretic use was nominally less in MADIT-II (76%) than in our observation (86%). More patients received betablockers, ACE-inhibitors and (at least for ICD patients) statins; antiarrhythmic use was substantially lower (4 vs. 15%). This might be attributable to the fact that our patients were included between 2003 and 2006 after the publication of MADIT-II, for which patients were included between 1997 and 2001. These data illustrate that patients from recent registries such as our observation might have a differential profile to randomized, controlled trials conducted previously. With the more frequent use of optimal medical therapy patient prognosis has been so much improved that absolute risks for cardiovascular events and total mortality might differ substantially between respective patients [2].

**Table 4:** Clinical characteristics of patients in the MADIT II trial

	**MADIT II ICD +**	**MADIT II ICD –**
	**(n = 742)**	**(n = 490)**
	Prior myocardial infarction more than 28 days ago and an EF ≤ 30%	
		
Age (years)	64 ± 10	65 ± 10
Female Gender (%)	16	15
LVEF (% ± SD)	23 ± 5	23 ± 6
Pharmacotherapy		
Betablockers (%)	70	70
ACE inhibitors	68	72
Antiarrhythmics	16	14
Diuretic	72	81
Statins	67	64
Mean follow-up		
Total mortality	14.2	19.8
HR Total mortality		
Heart failure related hospitalization	19.9	14.9

Legend: HR, hazard ratio; CI, confidence interval, EF, ejection fraction; LV, left ventricular.

### Survival benefit in perspective

Both MADIT-II and our observation document improvements in overall survival which may reach about 31% such as in MADIT-II [7]. This is also compatible with SCD-HeFT, which reported a risk reduction of 23% (HR 0.77; p = 0.007) and an absolute decrease in mortality of 7.2% after five years in the overall population. Patients in SCD-HeFT were included based on NYHA class II or III heart failure and a LVEF ≤ 35% and followed for a median of 45.5 months. In comparison MADIT reported a risk reduction of 54% for patients with prior MI, and LVEF ≤ 35% (absolute risk 38.6 vs. 15.8%) and a documented episode of asymptomatic unsustained ventricular tachycardia; and inducible, non suppressible ventricular tachyarrhythmia on electrophysiologic study [11]. Against this background the risk reduction of 48% in our cohort appears high. This might have been due to confounders that we were not able to fully account for. For example there were more patients with triple vessel disease in the control compared to the ICD group (60.1 vs. 44.3%; p = 0.006).

The difference to our data on those of MADIT II is reasonable given that patients with previous proof of arrhythmia were specifically included. None of the patients in the ICD group and 9.8% of the control group died of primary arrhythmia which illustrates a stronger risk reduction for arrhythmic death. Data are however hard to compare due to substantial differences in antiarrhythmic drug use. For example MUST reported a reduced 5 year rate of cardiac arrest or arrhythmic death by 27% (HR 0.73) among patients with CAD, a LVEF ≤ 40% and asymptomatic, not sustained ventricular tachycardia [16].

### Heart failure and inappropriate shocks

An increased risk for heart failure was documented in our observation and also in the MADIT-II trial (Tables 1 and 4). Recently an analysis of risk factors for recurrent heart failure in the MADIT-II trial was published [17]: Risk factors for a first HF hospitalization included treatment with an ICD (HR 1.31; p = 0.05), NYHA class > II (HR 1.95; p < 0.001), female gender (HR 1.38; p = 0.05), atrial fibrillation (HR 1.90; p = 0.001), QRS >120 ms (HR 1.41; p = 0.01), diabetes mellitus (HR 1.51; p = 0.003), heart rate ≥80 (HR 1.35; p = 0.04), diuretic therapy (HR 1.82; p < 0.001), and the presence of prerenal azotemia (blood urea nitrogen:creatinine >20; HR 1.45; p = 0.01). The occurrence of one HF event after enrolment was associated with a 2.8-fold (p < 0.001) increase in the risk of death, whereas after the occurrence of a second event there was a 6.7-fold (p < 0.001) increase in the risk of subsequent mortality.

Inappropriate shocks (those not due to ventricular fibrillation) were also more frequent in patients dying within the observational period (although not significant). This has been associated to the presence of atrial fibrillation (odds ratio 6.2) in a recent analysis of 549 patients with heart failure and ICDs [18]. In a Chinese single center study documenting 34 patients between 2005 and 2009 reasons for inappropriate ICD discharge were documented [19]. They found these to be again related mainly to supraventricular tachyarrhythmias, especially atrial fibrillation. We were however not able to verify this association because of the low number of cases in our observation.

### Limitations

These data reproduce those found in prior randomized controlled trials. Although this is encouraging a number of limitations have to be acknowledged in addition to the ones mentioned before: 1) This was a retrospective study with all limitations inherent in such designs such as: significant bias that may affect the assignment to the treated group or the control group, incompleteness of data which were not fully captured to answer the question under investigation, difficulty in assessing a temporal or even causal relationship, reliance on the quality of data available with no possibility of verification. 2) The set of data obtained for the patients under investigation was limited so that data interfering with the outcome such those on renal function, biomarkers or the presence of atrial fibrillation were not considered in the analysis. 3) We were not able to report on different causes of death (such as arrhythmic) and only report overall survival. 4) Third the study is small allowing no detailed analyses and multiple adjustments due to limited power. 5) There is a slight imbalance in the degree of heart failure, age and the number of vessels diseased which may not have been adequately accounted for. 6) We obtained no reasons why one group was offered an ICD and the other group not thought both had an indication for ICD use.

## Conclusions

Appreciating the potential limitations of retrospective studies we found that, in patients with ischemic heart disease and left ventricular dysfunction, typical for those in the community setting, ICD use was associated with an improved survival. The benefits were however offset by heart failure and inappropriate shocks which may justify the further research into more advanced devices such as biventricular pacemakers.

## Competing interests

None of the authors reports a conflict of interest with respect to the topic of the present manuscript.

## Author contributions

TA, PB and AC were responsible for the conception and design of the registry, RF for the statistical analysis. PB drafted the manuscript. JB, SH, CZ and UT revised the manuscript for important intellectual content. All authors read and approved the final manuscript.

## Availability of supporting data

None.
